# Correlation of Occupational Exposure to Carcinogenic Polycyclic Aromatic Hydrocarbons (cPAHs) and Blood Levels of p53 and p21 Proteins

**DOI:** 10.3390/biom12020260

**Published:** 2022-02-05

**Authors:** Saleh A. K. Saleh, Heba M. Adly, Imad A. Aljahdali, Abdullah A. Khafagy

**Affiliations:** 1 Biochemistry Department, Faculty of Medicine, Umm Al-Qura University, Makkah 21955, Saudi Arabia; saabdrabou@uqu.edu.sa; 2 Oncology Diagnostic Unit, Faculty of Medicine, Ain Shams University, Cairo 11435, Egypt; 3 Community Medicine and Pilgrims Healthcare Department, Faculty of Medicine, Umm Al-Qura University, Makkah 21955, Saudi Arabia; iajahdali@uqu.edu.sa (I.A.A.); aakhafagy@uqu.edu.sa (A.A.K.)

**Keywords:** carcinogenic polycyclic aromatic hydrocarbons (cPAHs), p53 protein, p21 protein, carcinogenesis, ambient air pollution, Makkah

## Abstract

Carcinogenic polycyclic aromatic hydrocarbons (cPAHs) are considered the most serious cancer risk. This study was conducted to assess the effect of acute exposure to cPAHs on cancer biomarker proteins p53 and p21 in occupational workers during the hajj season in Makkah. One hundred five participants were recruited, including occupational workers and apparently healthy individuals; air samples were collected using personal sample monitors to identify the subjects’ exposure to cPAHs. Quantitative analyses of benzo(a)anthracene (BaA), benzo(b)fluoranthene (BbF), benzo(a)pyrene (BaP), dibenzo(a,h)fluronathene (DBA), indeno(1,2,3-c,d)pyrene (IND) and chyresene (CRY) were carried out using the GC/Mass technique. Serum p53 and p21 proteins were analyzed using ELISA. The ambient air samples collected by the occupationally exposed group were more highly polluted by cPAHs, (90.25 ± 14.1) ng/m^3^, than those of the unexposed control groups, (30.12 ± 5.56) ng/m^3^. The concentration of distributive cPAHs was markedly more elevated in the air samples of the exposed group than in those taken from the non-exposed group. The study results demonstrated significant links between short-term exposure to cPAHs and serum p53 and p21 levels. Serum p53 and p21 proteins potentially influence biomarkers when exposed to ambient air cPAHs.

## 1. Introduction

Air pollution has become a pivotal issue for many countries. As an essential component of the climate system, environmental pollution is one reason for the global increase in attention given to climate change. Ambient air pollution in Saudi Arabia has become a focal point for a wide range of scientific, economic, industrial and social development concerns [1]. These concerns have arisen as a result of a significant increase in both stationary and mobile air pollution sources caused by growing industries and industrial areas, as well as the increased number of cars, buses and trucks. Poor urban air quality is still a challenge in many cities in Saudi Arabia, and solutions to control air pollutants remain insufficient [2,3].

The Makkah Al-Mokarramah region had a total population of 9,033,491 according to 2019 reports. Makkah, the holy city, has extraordinary characteristics. It has a population of approximately 1,700,000 and, during hajj and umrah seasons, it is estimated that 2,371,675 pilgrims visit Makkah, 75% of whom come from outside the country, in addition to about 3,000,000 umrah visitors each year [4]. This increase in visitors leads to a tremendous rise in the use of public transport, including buses, trucks and cars, leading to an elevated consumption of fuel and petrol products as well as increased concentrations of suspended dust, which influence air pollution emissions [5]. Additionally, more than 287,300 employees from both government and private groups are employed to deliver their services to pilgrims from all sectors, providing more than 215 services for pilgrims. Most of these labor forces work outdoors and are exposed to the acute pollution caused by the enormous number of vehicles, increased traffic and crowdedness [5,6]. Previous studies have reported the emissions from vehicles and trucks, fuel evaporation, dissolved petrol and dust are significant air pollutants in Makkah [7,8].

Cancer is a principal burden of disease worldwide. More than 19 million new cases are diagnosed each year around the world, and more than half of these patients eventually die from cancer [9]. Although Saudi Arabia has low cancer incidence rates, the prediction of its future incidence and mortality burden has been remarkable in the country. Based on GLOBOCAN 2020 data and statistics from the International Agency for Research on Cancer, by 2030 new cancer cases in Saudi Arabia are expected to increase by more than 50% [10]. Epidemiological studies led to the hypothesis that environmental factors enhance the progression of latent cancer to a clinical disease. Hence, there is an increased attention to the prospective factors that influence the incidence of this disease [11]. In this context, exposure to environmental pollution, food, alcohol and smoking is accused of contributing to this marked process [12].

Polycyclic aromatic hydrocarbons (PAHs), the principal chemical ingredients of petroleum, are widely spread in almost all environments [13]. PAHs are products of the incomplete combustion of organic compounds and are considered to be the most common and dangerous source of carcinogenic risk [14,15]. Carcinogenic PAHs (cPAHs) are compounds that can have serious health consequences due to their high tumorigenicity rates. cPAH exposure is a major area of interest within the field of carcinogenesis and environmental pollutants [16]. Many PAHs are considered to be potent human carcinogens, such as benzo[a]pyrene (BaP), dibenzo[a,l]pyrene (DBa,lP), benzo[a]anthracene (BaA), dibenzo[a,h]anthracene (DBA), benzo[b]fluoranthene (BbF), and indeno[1,2,3-c,d]pyrene (IND) [17,18,19,20,21]. The benzene pollution of ambient air is predominantly caused by buses, trucks and cars; therefore, the highest levels of contamination by these compounds are found near highways and high-traffic areas [22]. Several epidemiologic studies have documented the relationship between exposure to PAHs and the rising morbidity and/or mortality rates of respiratory diseases, cardiac diseases and cancer [23], and other reports indicated that the global proportion of lung cancer deaths attributed to outdoor ambient air pollution was about 14% in 2017 [24]. The PAHs require metabolic activation reactions prior to their genotoxic effects taking place, and research has recognized the critical role played by PAHs in the development of the abovementioned diseases and has documented that people exposed to urban pollution show increased levels of (PAH) DNA adducts. This supports the observation that a significant increase in genotoxicity and carcinogenicity can be caused by the presence of PAHs in the air [25,26]. Inhalation and/or absorption of cPAHs into the human body has the potential to produce free radicals and reactive oxygen species (ROS), which can oxidatively modify DNA and induce inflammation [27]. The process of ROS production is unconfirmed. There are three prospects for the derivation of radicals: inflammatory responses induced by particles; Fenton-radical-mediated processes, implying particle-associated transition metals; or redox-cycling processes coupled with metabolic reactions of xenobiotics [28].

The activities of the p53 gene have been comprehensively studied [29]. p53 is one of the key cancer genes and mutates in more than 50% of human malignancies. It has an influential role in microRNA regulation and participates in many biofunctions within the cells. Despite these carcinogenic properties, when the p53 protein has not mutated, it acts as a tumor-suppressing protein; it is a nuclear phosphoprotein that has an impact on the regulation of a wide range of reactions, such as genotoxic cellular stresses, leading to multiple responses including the stimulation of DNA repair, initiation of cell cycle arrest and apoptosis [30].

p53 protein synthesis in normal and unstressed cells is maintained at a lower level through ubiquitin-mediated proteolysis. TP53 gene mutations are common characteristics of the cancer genotype, which may lead to the disruption of the p53 protein’s standard response, resulting in tumorigenesis. Moreover, mutated p53 proteins can develop new tumorigenic functions, identified as gain-of-function activities (GOF), which help to induce and spread tumors in mouse models [31]. It can be shown that the overexpression of the p53 protein can reflect critical cellular and molecular events that lead to carcinogenesis. p53 overexpression in tumor tissue can be represented by increased levels of blood p53 protein [32], and the disequilibrium between cell proliferative activity and apoptosis triggers tumorigenesis [33]. Several studies explored the role of the p53 protein as an adjuvant monitoring and prognostic biomarker for follow-up cancer patients and/or a predictor of recurrence [34,35,36] in bladder, head, neck, esophagus and colorectal cancers [37,38]. As the p53 gene is implicated in carcinogenesis, it also has other regulatory functions in metabolic pathways such as glycolysis and oxidative phosphorylation [39].

The human p21 protein (also identified as wildtype activating factor-1/WAF1/CIP1) is a cyclin-dependent kinase (cdk) inhibitor, and the p53 protein is the chief regulator of its transcription. DNA damage drives the recognized p53 pathway, including an increase in p21 protein, which can arrest the cell cycle or stop its progression either in the G1, S or G2 phase [40]. p21 was found to be coupled with cellular sensitivity to transforming growth factor-beta (TGF-B) [41], which plays a key role in tumor progression, invasion and metastasis [42]. In addition to cell cycle arrest, p21 affects senescence events [43,44] and controls many cellular events such as the response to DNA damage and apoptosis. Thus, the influence of the p21 protein in carcinogenesis is fundamentally based on the status of the p53 protein [45]. In contrast, p21 can drive tumor development, causing cancer growth via the deceleration of accumulating DNA damage. The induction of p21 is essential for the promotion of carcinogenesis [46]; accordingly, p21 can act either as an oncogenic protein or a tumor suppressor, based on its cytoplasm or nucleus localization. This may increase the challenge to establish a proper equilibrium in which p21 would selectively obstruct cancer progression [47].

The induction of p53 and p21 proteins by PAHs in vitro was reported [48]. The blood levels of the p53 protein may indicate genotoxic stress and reflect exposure to many environmental pollutants, such as chromium, asbestos, PAHs, vinyl chloride, acrylonitrile, formaldehyde and ionizing radiation. Despite this, few studies have evaluated the effect of occupational exposure to cPAHs on the serum concentrations of the p53 protein [49,50]. While the mechanism of PAHs’ carcinogenicity remains undefined, their correlations with p53 gene mutation in patients with breast and lung cancers were reported [51,52]. Although extensive research has been carried out on the health effect of PAHs, there are few reports on the health effects of occupational exposure to ambient air contaminants in Saudi Arabia, particularly acute short-term exposure in Makkah.

This study was conducted to assess the effect of acute exposure to cPAHs on cancer biomarker proteins p53 and p21 in occupational workers during the hajj season in Makkah. To the best of our knowledge, this is the first study to address this health problem in Makkah.

## 2. Materials and Methods

### 2.1. Study Population

This study included 60 individuals, comprised of employees in a range of sectors who deliver their services to pilgrims during the hajj season, including bus and truck drivers, police officers, Islamic affairs and pilgrim guidance. These labor forces are working outdoors for ≥8 h/day, exposing them to vehicles, high levels of traffic and large crowds. The control group is comprised of 45 apparently healthy individuals spending >80% of daily time indoors or working in a suburban area that is not exposed to petrol compounds.

All participants in this study were volunteers of matched socio-economic status, non-smokers and had no history of alcohol or drug abuse. Data were recorded for all individuals, including clinical history, age, working time, previous job, family history and socioeconomic status. Informed consent was collected from all participants before the study.

### 2.2. Assessment of Exposure to External cPAHs

Exposure to external cPAHs was assessed by personal air sampling GC-TurboMatrix ATD 110 (Perkin Elmer, Waltham, MA, USA). Three personal air sampler monitors were distributed to each studied subject and air samples were collected each day for three successive days after their working shift in the hajj season. The personal air sampler monitors contain air tubes used for different air toxins varying in volatility from propylene to hexachlorobuta-1,3-diene, and may collect polar and nonpolar organic compounds. The tube specifications included: Tube type was glass; Sorbent(s) were Carbograph 1TD/Carboxen 1003 (air toxics); Target analytes C2/3 to n-C20. These specifications were used to cover this range of analytes in accordance with USA EPA Method TO-17.

The quantitative analyses were performed to measure the levels of cPAHs by using a Perkin Elmer GC/Mass TurboMatrix ATD-600 (Perkin Elmer, Waltham, MA, USA) according to the manufacturer’s instructions. ATD TurboMatrix techniques utilized a purify time of 5 min, with column flow rate of 2 mL/min, and an outlet split of 6 mL/min. The valve and transfer line temperatures were placed at 270 °C and 290 °C, correspondingly, and the trap temperature was placed from 10 °C to 385 °C with a 10 min hold. An Rxi-5Sil MS capillary GC column with a 5 m Integra Guard column, 0.25 mm ID, 30 m length, and 0.25 mm film thickness was used. Research-grade helium gas (99.9995%) and ultra-zero air were used. The GC oven temperature was held at 35 °C for 2 min and increased to 190 °C at 6 °C/min, then increased to 310 °C at 28 °C/min and held for 8 min. The quadrupole, ion source and transfer line temperatures were 176 °C, 290 °C and 290 °C, respectively. Scan spectra were gathered at a rate of 22. Ions were observed from 35 to 300 m/z. PAHs were isolated with 10 mL of DCM/n-hexane (1:1), fractionated by column chromatography, and eluted with 20 mL of n-hexane/dichloromethane (1:1, *v*:*v*) A 2 L sample of the extract was inserted into a GC-Mass Clarus 600. The Gas Chromatography was adjusted with a diluted standard solution of different PAH compounds (Supelco, Inc., Bellefonte, PA, USA) and the levels of the following cPAHs: benzo(a)anthracene (BaA), benzo(b)fluoranthene (BbF), benzo(a)pyrene (BaP), dibenzo(a,h)fluronathene (DBA), Indeno(1,2,3-c,d)pyrene (IND) and chyresene (CRY) were measured. Chromatographic peaks were incorporated using NIST Mass Spectral Library.

### 2.3. Blood Sample Preparation and Storage

Blood samples were collected from each participant at the end of their working shift using plain tubes and allowed to clot. Blood sample tubes were centrifuged; the serum was separated from the samples, refrigerated at −20 °C and analyzed within one week.

### 2.4. Measurement of Serum p53 and p21 Protein Concentrations

In all participants, the serum levels of the p53 protein were analyzed in the collected serum samples using Human p53 ELISA kits (Sigma-Aldrich Chemie GmbH, Kappelweg, Germany) according to the manufacturer’s instructions. The serum levels of p21 protein were analyzed by Human P21 ELISA Kit (Proteintech Group, Inc., Rosemont, IL, USA) according to the manufacturer’s instructions. The overall intra-assay coefficient of the variant of independent assays was determined to evaluate the reproducibility of experiments.

### 2.5. Statistical Evaluations

Standard analytical methods were applied with each assay, and the conditional regression method was used to calculate the odd ratios and 95% confidence intervals for all exposure intervals. The significance level was established using a *p* value of <0.05. IBM SPSS Statistics v. 26 (IBM, New York, NY, USA) was used for the statistical analysis.

## 3. Results

The number and mean age of the occupationally exposed group and unexposed control group are shown in Table 1. Table 2 showed that the ambient air samples collected by the occupationally exposed group were more highly polluted by cPAHs, (90.25 ± 14.1) ng/m^3^, than those of the unexposed control group, (30.12 ± 5.56) ng/m^3^; the difference was statistically significant (*p* < 0.001).

The mean concentrations of individual cPAHs analyzed in the ambient air samples of the occupationally exposed group, BaA, BbF, BaP, DBA, IND and CRY, were significantly higher than in the unexposed control group (*p* < 0.001). The mean serum levels of p53 and p21 proteins were significantly higher in the occupationally exposed group than in the unexposed control group (*p* < 0.05), as shown in Table 3.

**Figure 1:**
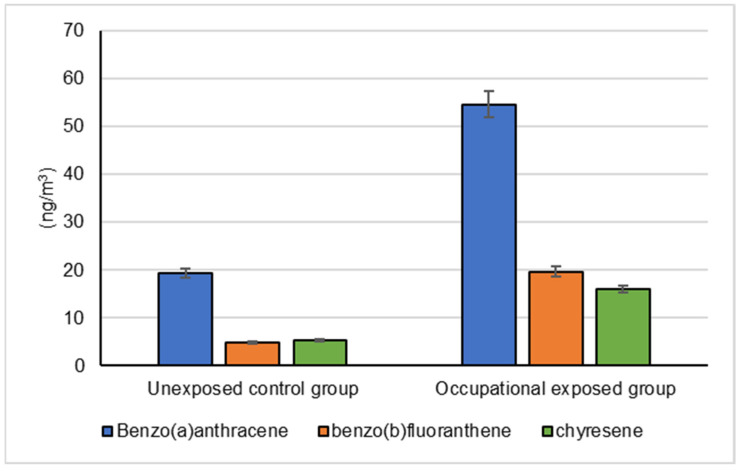
Mean levels of benzo(a)anthracene, benzo(b)fluoranthene and chyresene in the collected air samples by the occupationally exposed group and the unexposed control group, (*p* < 0.001).

**Figure 2:**
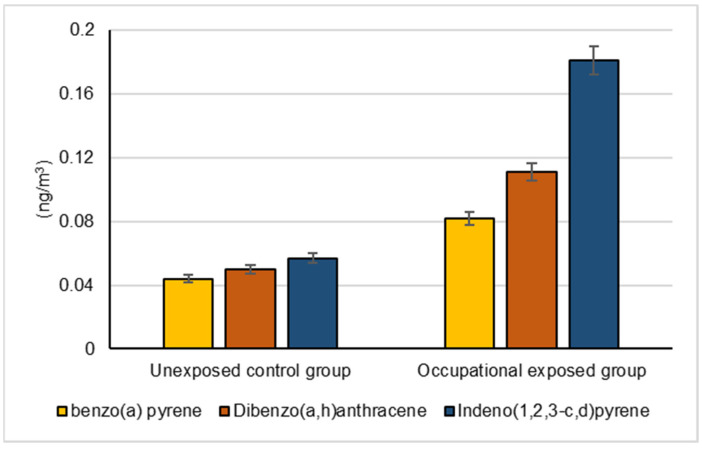
Mean levels of benzo(a)pyrene, dibenzo(a,h)anthracene and indeno(1,2,3-c,d)pyrene in the collected air samples by the occupationally exposed group and the unexposed control group, (*p* < 0.001).

The mean levels of BaA, BbF and CRY in the collected ambient air samples that were collected by the occupationally exposed group and unexposed control groups are represented in Figure 1. The mean levels of BaP, DBA and IND in the collected ambient air samples that were collected by the occupationally exposed group and the unexposed control group are illustrated in Figure 2.

**Figure 3:**
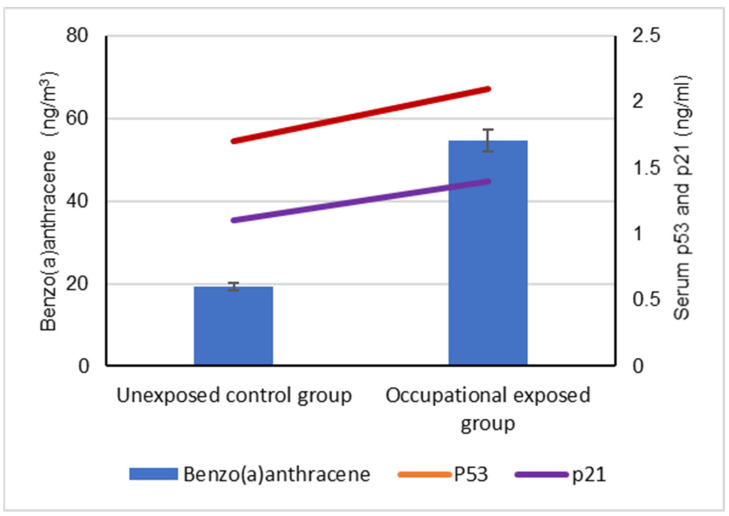
Correlation between atmospheric exposure to benzo(a)anthracene and serum levels of p53 and p21 proteins, (*p* < 0.05).

**Figure 4:**
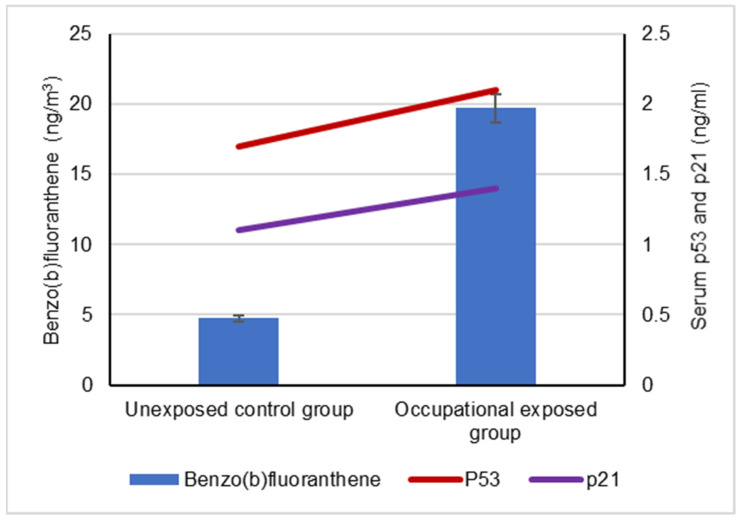
Correlation between atmospheric exposure to benzo(b)fluoranthene and serum levels of p53 and p21 proteins, (*p* < 0.05).

**Figure 5:**
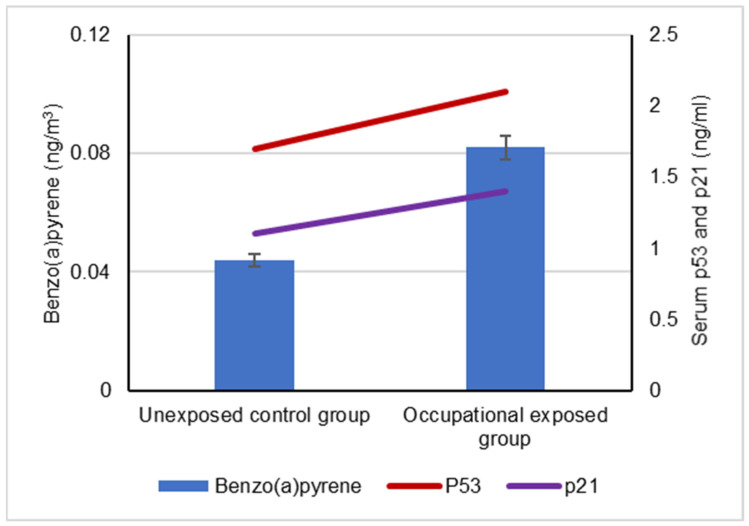
Correlation between atmospheric exposure to benzo(a)pyrene and serum levels of p53 and p21 proteins, (*p* < 0.05).

**Figure 6:**
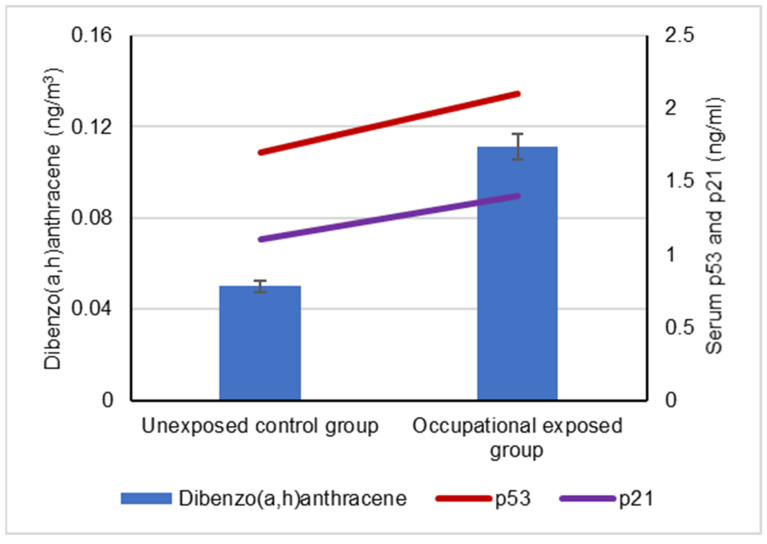
Correlation between atmospheric exposure to dibenzo(a,h)anthracene and serum levels of p53 and p21 proteins, (*p* < 0.05).

**Figure 7:**
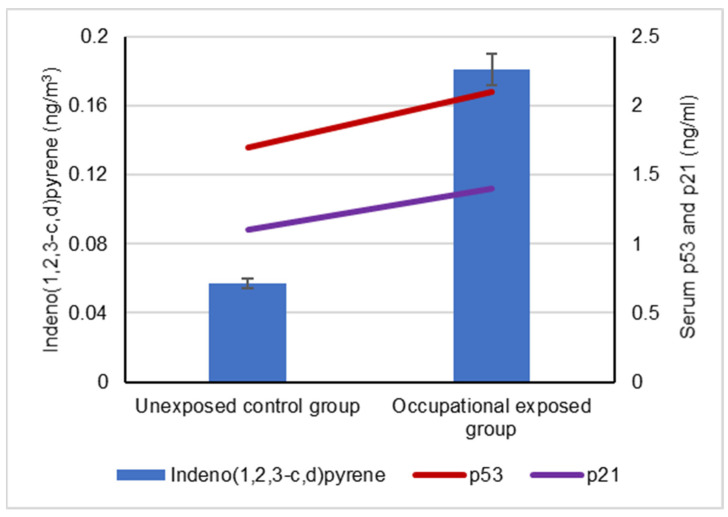
Correlation between atmospheric exposure to indeno(1,2,3-c,d)pyrene and the serum levels of p53 and p21 proteins, (*p* < 0.05).

**Figure 8:**
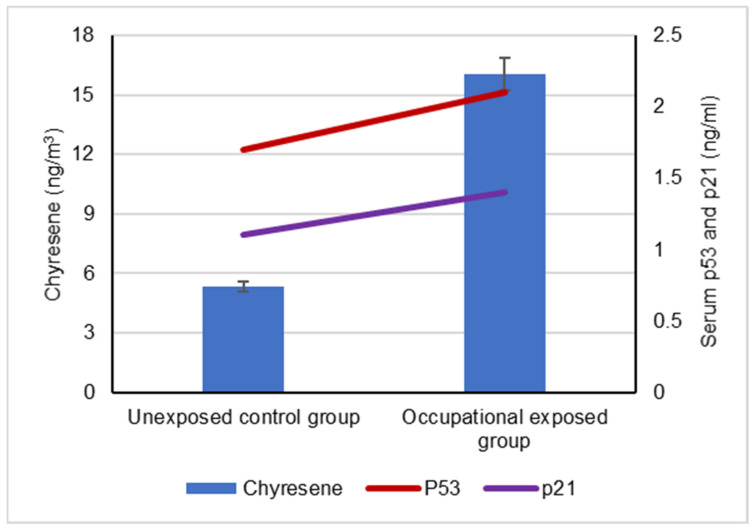
Correlation between atmospheric exposure to chyresene and the serum levels of p53 and p21 proteins, (*p* < 0.05).

**Figure 9:**
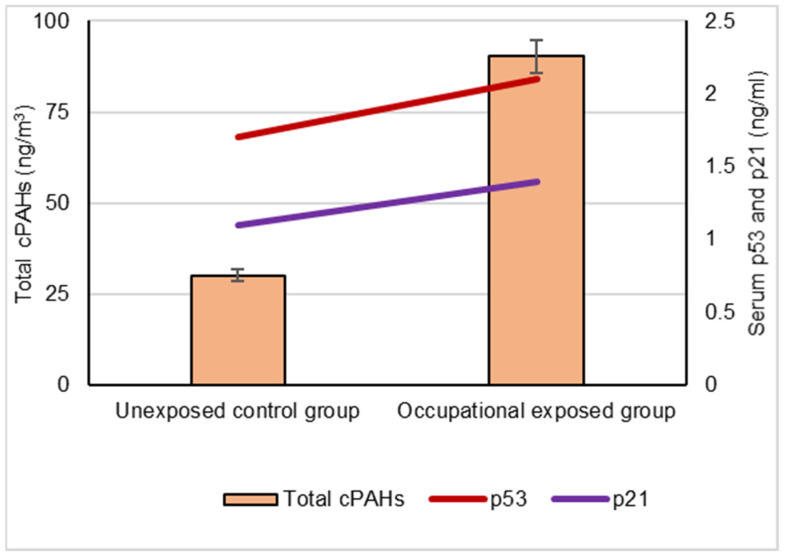
Correlation between atmospheric exposure to total cPAHs and the serum levels of p53 and p21 proteins, (*p* < 0.05).

Our results showed a significant difference between the serum levels of the p53 protein in occupational workers exposed to cPAHs (2.2 ± 0.5 ng/mL) and (1.7 ± 0.3 ng/mL) and the control group, indicating a positive correlation with increased cPAH exposure as well as the individual cPAHs analyzed: BaA, BbF, BaP, DBA, IND and CRY (*p* < 0.05), as shown in Figure 3, Figure 4, Figure 5, Figure 6, Figure 7, Figure 8 and Figure 9. Serum p21 protein was significantly more elevated in the occupationally exposed group than the unexposed control group (*p* < 0.05). Additionally, exposure to the individual cPAHs analyzed, BaA, BbF, BaP, DBA, IND and CRY, showed an increased level of serum p21, (*p* < 0.05), as shown in Figure 3, Figure 4, Figure 5, Figure 6, Figure 7, Figure 8 and Figure 9.

## 4. Discussion

PAHs are formed as products of incomplete fossil fuel combustion, biomass burning or solid waste incineration [53,54]. They are semi-volatile and ever-present persistent organic pollutants (POPs) that can be transported away from their emission source in air masses either as gas molecules or bound particulate matter [55,56,57]. Ingestion and inhalation are the most common way for humans to be exposed to PAHs [17].

In the current study, we analyzed the exposure effect of cPAHs in ambient air on the serum levels of p53 and p21 proteins among the exposed group, consisting of sector employees who deliver their services to pilgrims during the hajj season in Makkah, including bus and truck drivers, police officers, Islamic affairs and pilgrim guidance. These participants work outdoors for ≥8 h/day, exposing them to vehicles and high levels of traffic. The reports on the concentrations of p53 and p21 proteins in serum of occupationally exposed workers are contradictory and are primarily focused on p53 protein levels. In the current study, the outdoor working group were subjected to higher levels of total cPAHs compared to the unexposed control group; the mean levels of total cPAHs in the collected air samples were (90.25 ± 14.1 ng/m^3^) and (30.12 ± 5.56 ng/m^3^) for the occupationally exposed group and unexposed control group, respectively. Additionally, all the individual cPAHs measured, benzo(a)anthracene, benzo(b)fluoranthene, benzo(a) pyrene, dibenzo(a,h)anthracene, indeno(1,2,3-c,d)pyrene and chyresene, were found to be higher in air samples collected by the occupationally exposed groups than the unexposed control groups.

Environmental contaminants can alter genome stability via epigenetic modifications [58]. Based on large-scale cohort studies, there is a sensible basis for the attention given to urban air pollution, as it may increase cancer risk, particularly in association with other risk factors such as active and passive smoking and occupational exposures [27,59,60]. Several epidemiological studies have documented the quantifiable links between occupational ambient contaminant exposure and the increased risk of cancer, particularly lung cancer [61].

The mutation of the p53 gene affects its capability to stimulate tumor-suppressing pathways, leading to tumorigenesis. p53 plays a major role sustaining genomic stability and homeostasis. The p53 protein governs the expression of its downstream effector genes, the expressions of which are linked to key cellular processes such as DNA repair, cell cycle control and apoptosis. The overexpression of the p53 protein is the cellular response to genotoxic stress [62,63]. The serum level of p53 and p21 proteins were evaluated in this study as hypothesized cancer risk biomarkers. A statistical analysis of serum p53 protein level and atmospheric exposure to cPAHs in the occupationally exposed group showed positive correlations between p53 protein concentration and total cPAHs, as well as a positive correlation with individual cPAH exposure. Serum p21 protein levels were greater in occupational workers exposed to cPAHs than in the unexposed control group. It has been suggested that exposure to complex PAH mixtures may stimulate and/or induce coactive or opposed impacts on the genotoxic features of PAHs, which may affect the expression of p53 and p21 proteins. Thus, concentrations of individual PAHs may have another role which affects the expression of these proteins [64]. Previous studies revealed that the p53 protein may accumulate as a response to cPAH exposure [65]. Consequently, elevated serum levels of p53 protein in the exposed occupational workers is attributed to an increased exposure to cPAHs in the occupational workers, and the serum p53 level can indicate exposure to cPAHs.

These results corroborate previous studies which declared a positive relationship between exposure to genotoxic agents and concentration of p53 protein [66,67]. Labib et al. (2012) found that rats orally exposed to benzo[a]pyrene had increased levels of lung mRNA cancer-related genes and most of them were engaged in the tumor-suppressing p53 pathway as cell cycle arrest and apoptosis [68]. In addition, p53 ribonucleotide reductase has been endorsed as a biomarker for benzo[a]pyrene exposure [69].

Some studies reported that low-molecular-weight PAHs, such as fluoranthene and benzo[c]phenanthrene, can inhibit enzymes that are involved in PAHs’ metabolic activation, leading to a reduction in the potential and genotoxic effects of some cPAHs [38]. However, our results demonstrated a positive correlation between short periods of cPAH exposure and a significant increase in serum p53. This finding is reinforced in the report by Borska et al. (2009), which showed a significantly elevated level of p53 in the plasma of individuals with increased cPAH exposure during a short period of time. They revealed that a significant increase in plasma p53 occurred as a result of skin exposure to cPAHs in psoriatic patients who utilized coal tar for 1–4 weeks as a treatment [37]. Our results indicated increased cPAH concentrations in air samples during the hajj season in Makkah, which may correlate with an increased cancer risk for occupational workers who are exposed to high-traffic areas during the hajj season.

## 5. Conclusions

The results of this study demonstrate significant links between exposure to cPAHs and serum p53 and p21 levels, as short-term exposure to highly cPAH-contaminated ambient air may result in an increasing amount of p53 and p21 proteins. Serum p53 and p21 proteins may influence biomarkers when exposed to ambient air cPAHs; however, further studies are required with a follow-up period, particularly for long-term exposure, to evaluate the validity and specificity of this finding.

## Figures and Tables

**Table 1:** Characteristics of studied groups.

Studied Groups	Occupationally Exposed Group	Unexposed Control Group	*p*-Value
	Mean ± SD	Mean ± SD	
Number	60	45	
Age (y)	34.5 ± 4.2	36.5 ± 5.1	>0.05

**Table 2:** Mean levels of cPAHs in the collected air samples in the occupationally exposed group and the unexposed control group.

Studied Groups	Occupationally Exposed Group	Unexposed Control Group	*p*-Value
	Mean ± SD	Mean ± SD	
BaA (ng/m^3^)	54.63 ± 8.12	19.30 ± 4.03	<0.001
BbF (ng/m^3^)	19.7 ± 2.44	4.75 ± 1.01	<0.001
BaP (ng/m^3^)	0.082 ± 0.032	0.044 ± 0.006	<0.001
DBA (ng/m^3^)	0.111 ± 0.025	0.050 ± 0.009	<0.001
IND (ng/m^3^)	0.181 ± 0.059	0.057 ± 0.009	<0.001
CRY (ng/m^3^)	16.05 ± 3.68	5.32 ± 0.88	<0.001
Total cPAHs (ng/m^3^)	90.25 ± 14.1	30.12 ± 5.56	<0.001

BaA, benzo(a)anthracene; BbF, benzo(b)fluoranthene; BaP, benzo(a) pyrene; DBA, dibenzo(a,h)anthracene; IND, indeno(1,2,3-c,d)pyrene; CRY, chyresene.

**Table 3:** The mean serum levels of p53 and p21 proteins.

Studied Groups	Occupationally Exposed Group	Unexposed Control Group	*p*-Value
	Mean ± SD	Mean ± SD	
Serum p53 protein (ng/mL)	2.2 ± 0.5	1.7 ± 0.3	<0.05
Serum p21 protein (ng/mL)	1.4 ± 0.3	1.1 ± 0.2	<0.05

## Data Availability

The data presented in this study are available on request from the corresponding author.
